# Evaluation of Global DNA Methylation and Gene Expression of *Izumo1* and *Izumo1r* in Gonads after High- and Low-Dose Radiation in Neonatal Mice

**DOI:** 10.3390/biology10121270

**Published:** 2021-12-03

**Authors:** Akifumi Nakata, Keisuke Sato, Yohei Fujishima, Valerie Goh Swee Ting, Kanade Nakayama, Kentaro Ariyoshi, Chizuru Tsuruoka, Yi Shang, Daisuke Iizuka, Shizuko Kakinuma, Hideaki Yamashiro, Tomisato Miura

**Affiliations:** 1 Faculty of Pharmaceutical Sciences, Hokkaido University of Science, Sapporo 006-8585, Japan; sato-k@hus.ac.jp (K.S.); 117a175@stu.hus.ac.jp (K.N.); 2 Department of Risk Analysis and Biodosimetry, Institute of Radiation Emergency Medicine, Hirosaki University, Hirosaki 036-8564, Japan; yohei.fujishima@med.tohoku.ac.jp; 3 Department of Radiobiology, Singapore Nuclear Research and Safety Initiative, National University of Singapore, Singapore 138602, Singapore; snrgstv@nus.edu.sg; 4 Graduate School of Health Sciences, Hirosaki University, Hirosaki 036-8564, Japan; 5 Center for Integrated Science and Humanities, Fukushima Medical University, Fukushima 960-1247, Japan; ariyoshi@fmu.ac.jp; 6 Department of Radiation Effects Research, National Institute of Radiological Sciences (NIRS), National Institutes for Quantum Science and Technology (QST), Chiba 263-8555, Japan; tsuruoka.chizuru@qst.go.jp (C.T.); shang.yi@qst.go.jp (Y.S.); iizuka.daisuke@qst.go.jp (D.I.); kakinuma.shizuko@qst.go.jp (S.K.); 7 Graduate School of Science and Technology, Niigata University, Niigata 959-2181, Japan; hyamashiro@agr.niigata-u.ac.jp

**Keywords:** dose-rate effect, irradiation before sexual maturity, global DNA methylation, testis, ovary, *Izumo1*, *Izumo1r*

## Abstract

**Simple Summary:**

Other than DNA damage, it has become clear that ionizing radiation can also induce epigenetic effects. Thus, intergenerational effects from chronic low-dose exposures are possible concerns. It is important to elucidate the effects of radiation on germ cell maturation, fertilization and embryonic development. We analyzed global DNA methylation patterns in the testes and expression of *Izumo1* in the testes and *Izumo1r* (*Izumo1* receptor) in the ovaries of mice after neonatal acute high-dose radiation (HDR) and chronic low-dose radiation (LDR). The results of global DNA methylation patterns in the testis showed that the LDR group maintained its DNA methylation level, while the HDR group showed decreased DNA methylation levels with age. The expression level of *Izumo1* in the testis recovered after the end of irradiation in the LDR group, while it remained low regardless of age in the HDR group. Although the expression of *Izumo1r* in the ovary decreased with age in the LDR group, it remained low in the HDR group. Our results indicate that LDR can induce different DNA methylation patterns, and both low- and high-dose radiation before sexual maturity might affect gametogenesis and fertility.

**Abstract:**

The intergenerational effects from chronic low-dose exposure are matters of concern. It is thus important to elucidate the radiation-induced effects of germ cell maturation, fertilization and embryonic development. It is well known that DNA methylation levels in CpG sites in gametes are reprogrammed in stages during their maturity. Furthermore, the binding of *Izumo* on the surface of sperm and *Juno* on the surface of oocytes is essential for fertilization. Thus, there is a possibility that these genes are useful indicators to evaluate fertility in mice after irradiation exposure. Therefore, in this study, we analyzed global DNA methylation patterns in the testes and gene expression of *Izumo1* and *Izumo1r* (*Juno*) in the gonads of mice after neonatal acute high-dose ionizing radiation (HDR) and chronic low-dose ionizing radiation (LDR). One-week-old male and female mice were irradiated with a total dose of 4 Gy, with acute HDR at 7 days at a dose rate of 30 Gy/h and LDR continuously at a dose rate of 6 mGy/h from 7 to 35 days. Their gonads were subsequently analyzed. The results of global DNA methylation patterns in the testes showed that methylation level increased with age in the control group, the LDR group maintained its DNA methylation level, and the HDR group showed decreased DNA methylation levels with age. In the control group, the gene expression level of *Izumo1* in the testis did not show age-related changes, although there was high expression at 100 days of age. However, in the LDR group, the expression level recovered after the end of irradiation, while it remained low regardless of age in the HDR group. Conversely, gene expression of *Izumo1r* (*Izumo1* receptor) in the ovary decreased with age in the control group. Although the gene expression of *Izumo1r* decreased with age in the LDR group, it remained low in the HDR group. Our results indicate that LDR can induce different DNA methylation patterns, and both high- and low-dose radiation before sexual maturity might affect gametogenesis and fertility.

## 1. Introduction

As ionizing radiation can cause a variety of health effects, it is necessary to reduce radiation exposure and radioactive material contamination to as low as possible. However, radiation exposure can also occur due to occupational and medical exposures as well as from nuclear power plant accidents such as in Chernobyl and Fukushima. It is known that radiation exposure can cause germ cell death, differentiation, termination of development and possibly infertility. Another important effect is the damage to the reproductive function of germ cells, which might affect several generations in the form of malformations and other abnormalities and diseases through the germ cells. Male germ cells in the testes are known to undergo cell death and increased sperm malformation after high-dose radiation [1,2,3]. Conversely, in the ovary, the total number of follicles is depleted in a dose-dependent manner by high-dose radiation exposure [4]. Decreased litter size is also commonly seen after high-dose radiation [5,6].

Recently, it has become clear that ionizing radiation can induce a wide range of DNA damage, including base lesions [7], DNA strand breaks [8], cross-linking of DNA [9,10] and epigenetic effects [11]. DNA methylation is one of the major epigenetic mechanisms that protect the stability of the genome in the cell [12], including regulation of gene expression [13] and chromatin structure [14]. In mammals, DNA methylation is also important for appropriate cellular development and differentiation [15], and is involved in various diseases such as cancer [16] and imprinting disorders [17].

In embryogenesis and postnatal development in the offspring, the sperm and egg are required to reset the appropriate genome-wide epigenetic information during the differentiation of germ cell lineages [18,19]. Several studies have demonstrated that imbalances in DNA methylation of the genome in gametes are associated with infertility [20,21,22,23,24] or generate imprinting abnormalities in the offspring [25,26,27]. Moreover, radiation-induced global DNA methylation in various organs in sexually mature mice has been reported to change in a sex- and tissue-specific and dose-dependent manner [28,29,30,31,32]. Among them, in the testes, sexually mature mice collected at 6 months after irradiation showed a similar level of global methylation as compared to the control group [28]. On the other hand, it has been shown that the expression of DNA methyltransferase 1 and histone deacetylase 1 in the testis was downregulated by irradiation [33], suggesting that it may affect DNA methylation patterns. To the best of our knowledge, few studies were made to evaluate the long-term effects of radiation on gonads before, during and after sexual maturity. In addition, the gonad gene expression and epigenetic changes in mice irradiated before sexual maturity remain unknown.

When considering radiation effects on reproduction and development, it is necessary to elucidate not only gametogenesis and embryogenesis, but also fertilization capacity. In mammalian fertilization, a sperm surface protein, Izumo sperm–egg fusion 1 (*Izumo1*) [34] and its binding partner on the surface of oocytes, *Izumo1r* (sperm–egg fusion protein *Izumo1* receptor/Juno) [35] form an intercellular bridge. The sperm from *Izumo1* knockout mice has normal morphology and physiology, but fertilization does not occur between them and normal eggs [34,36]. Similarly, eggs from *Izumo1r*-deficient mice appear morphologically normal, but fertilization also does not occur with normal sperm [35]. Therefore, there is a possibility that these genes are useful as indicators to evaluate fertility in mice.

Previously, we have reported the increased risk of thymic lymphoma, B-cell lymphoma, liver cancer and lung cancer in mice after radiation exposure at infancy [37,38,39]. However, understanding the behavior of epigenetics and gene expressions related to fertilization has important implications for reproductive health and transgenerational effects, as these effects after long-term exposure to low-dose radiation are not as well studied in mice irradiated before sexual maturity. In this study, we examined global DNA methylation patterns in mouse testes after neonatal acute high-dose ionizing radiation (HDR) and chronic low-dose ionizing radiation (LDR). Moreover, expression levels of genes involved in gamete fusion in gonads (*Izumo1*, *Izumo1r*) were also analyzed.

## 2. Materials and Methods

### 2.1. Animals and Irradiation

F1 hybrid B6C3F1/Crlj mice (C57BL/6NCrlCrlj × C3H/HeNCrlCrlj, Charles River Laboratories, Kanagawa, Japan) were used in this study. Mice were housed five in a cage in specific-pathogen-free conditions on a 12 h light/dark cycle, at 23 ± 2 °C with 50 ± 10% humidity, on a standard laboratory diet (MBR-1; Funabashi Farm Co., Ltd., Chiba, Japan) with water ad libitum. A total of n = 30, 1-week-old, mice were separated into three groups, each with n = 10 mice, so that there were 5 females and 5 males in each group: (i) sham (non)-irradiated; (ii) acute HDR; and (iii) chronic LDR. In the HDR group, male and female mice were acutely irradiated with a ^137^Cs γ-irradiator (Gammacell 40; Nordion International, Ottawa, Canada; 57.35 TBq) at a dose rate of 0.47 Gy/min, with an estimated whole-body absorbed dose of 4 Gy. In the LDR group, male and female mice were chronically irradiated with a ^137^Cs γ-irradiator (Pony Industry Co., Ltd., Osaka, Japan; 1.11 TBq) at a dose rate of 0.1 mGy/min from 7 to 35 days, with the same estimated whole-body absorbed dose of 4 Gy. Control mice were sham treated. The experiment was terminated when mice of ages 35 and 100 days old were sacrificed. Gonads were collected and immediately frozen in liquid nitrogen, and stored at −80 °C until use. All experimental procedures were conducted according to the Guidelines for Animal Welfare and Experimentation of the National Institutes for Quantum Science and Technology of Japan. All mice experiments were approved by the Institutional Animal Care and Use Committee of the National Institutes for Quantum Science and Technology (approval no. 17-1008).

### 2.2. Global DNA Methylation Assessment

Genomic DNA was extracted from mice gonads using the QIAamp DNA Micro Kit (Qiagen, Hilden, Germany). Outlier samples with a low amount of DNA were excluded in global DNA methylation analysis. Global methylation levels were measured using a global DNA methylation (5 mC) Colorimetric ELISA Easy Kit (EpiGentek, Farmingdale, NY, USA) in accordance with the manufacturer’s instruction. GloMax Discover (Promega, Madison, WI, USA) was used to measure the amount of methylated DNA by reading the absorbance at 450 nm. A standard curve was used to quantify the absolute amount of methylated DNA (percentage of 5 mC) in the total genomic DNA. Each sample was assessed in duplicate.

### 2.3. Measurement of mRNA Levels by Quantitative RT-PCR

Total RNA from gonads was extracted using the RNeasy Mini Kit (Qiagen). RNA was reverse transcribed to cDNA using the High-Capacity cDNA Reverse Transcription Kit (Thermo Fisher Scientific, Vilnius, Lithuania). Quantitative RT-PCR analysis was performed using the ABI 7500 Fast Real-Time PCR System (Applied Biosystems Pleasanton, USA). TaqMan gene expression assays (Applied Biosystems) for Izumo sperm–egg fusion 1 (*Izumo1*) (Mm01205584_m1), IZUMO1 receptor, JUNO (*Izumo1r*) (Mm00491664_m1) and glyceraldehyde 3-phosphate dehydrogenase (*Gapdh*) (Mm99999915_g1), and TaqMan Fast Universal PCR Master Mix (Applied Biosystems, Vilnius, Lithuania) were used for reaction mixtures. Glyceraldehyde-3-phosphate dehydrogenase (*Gapdh*) was used as the endogenous housekeeping control gene.

### 2.4. Statistics

All results are presented as the median with 95% confidence interval unless otherwise specified. Normality was verified using Shapiro–Wilk test. Outliers were examined using the Smirnov–Grubbs test. All variables were assessed using the Kruskal–Wallis test, followed by Dunn’s multiple comparison test with Benjamini–Hochberg *p*-value adjustment. The results were considered statistically significant if *p*-values < 0.05 were obtained. All statistical analyses were performed using the R version 4.1.0 (R Development Core Team, Vienna, Austria) [40].

## 3. Results

### 3.1. Global DNA Methylation in the Testis

**Figure 1:**
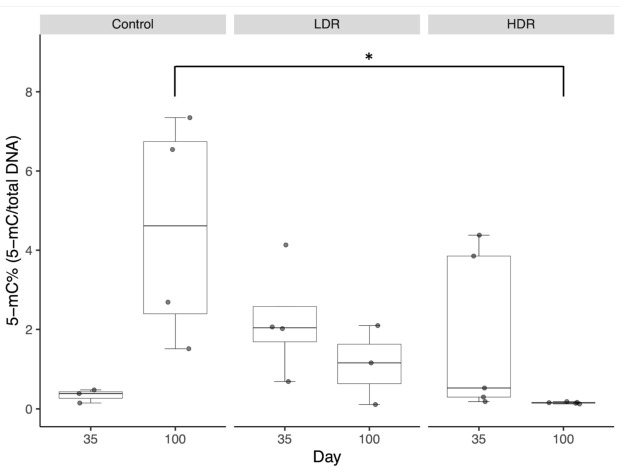
Radiation-induced changes in global DNA methylation patterns in the testis. ∗ Significant difference at *p* < 0.01. Sample medians are shown by the center line and crosses, respectively. Box edges represent the 25th and 75th percentiles.

To determine the radiation-induced changes in global DNA methylation patterns in the testis over a period of time, we analyzed the status of global DNA methylation of animals subjected to HDR and LDR after completion of irradiation. As the ovaries of irradiated mice were very small, global DNA methylation analysis was not possible. In this study, global DNA methylation analysis was performed only in testis. The global DNA methylation status was somewhat heterogeneous, but there was a consistent tendency among the groups (Figure 1).

The methylation level in the control mice increased from 0.33 (−0.09–0.76) at the age of 35 days to 4.52 (−0.02–9.07) at the age of 100 days. The methylation level of 35-day-old mice in the LDR group, after the end of irradiation, was higher than that of 35-day-old mice in the control group (2.22 [−0.04–4.49]), but the difference was not significant. Then, it decreased to 1.12 (−1.35–3.59) at 100 days of age. The median methylation levels at 35 days of age in the HDR group was high (1.85 [−0.74–4.43]), though there were a range of values in the HDR group. In the 100-day-old mice, the HDR group showed significantly lower levels compared to the control group (0.15 [0.13–0.18], *p* < 0.01).

### 3.2. The Gene Expression Profiles of Izumo1 and Izumo1r in the Gonads

**Figure 2:**
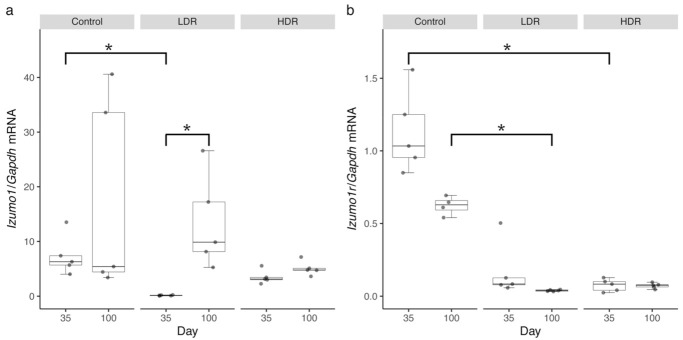
Expression levels of *Izumo1* (**a**) and *Izumo1r* mRNA (**b**) in male and female mice following radiation exposure. *Gapdh* was used as the endogenous housekeeping control. * Significant difference at *p* < 0.05. Sample medians are shown by the center line and crosses, respectively. Box edges represent the 25th and 75th percentiles.

The expression profiles of *Izumo1* in the testes following radiation exposure are shown in Figure 2. The expression of *Izumo1* mRNA increased with age in both control and LDR groups. In the control group, testicular *Izumo1* mRNA levels were 7.38 (2.86–11.90) at 35 days of age and 17.48 (−4.97–39.94) at 100 days of age. The LDR group showed significantly downregulated expression at the age of 35 days (0.14 [0.05–0.23]), which was after the end of irradiation, compared to the control group at 35 days (7.38 [2.86–11.90]) (*p* < 0.05). The expression of *Izumo1* in the LDR group was significantly upregulated from 0.14 (0.05–0.23) at 35 days of age to 13.41 (2.75–24.08) at 100 days of age (*p* < 0.05). However, the *Izumo1* transcript exhibited a marked decrease in the HDR group, which remained low with age.

Moreover, *Izumo1r* expression levels in the ovaries tended to decline with age in the control group. At 35 days of age, *Izumo1r* mRNA level was 1.13 (0.78–1.48), while at 100 days of age, it was 0.62 (0.52–0.72). Similarly, despite the low expression levels of the LDR group, *Izumo1r* transcript levels showed a decrease with age. The level of *Izumo1r* mRNA was 0.17 (−0.06–0.40) at 35 days of age, whereas it was 0.04 (0.03–0.05) at 100 days of age. *Izumo1r* expression levels remained low in HDR group as the median values for mRNA expressed at 35 days of age was 0.08 (0.02–0.13) and at 100 days of age was 0.07 (0.05–0.10). *Izumo1r* gene expression at 100 days of age was also significantly downregulated in the LDR than the control group (*p* < 0.05), but not significantly downregulated in the HDR group. On the other hand, *Izumo1r* gene expression at 35 days of age was more significantly downregulated in the HDR than the control group (*p* < 0.05), but not in the LDR group.

## 4. Discussion

The irradiated mice had fewer sperm in the seminiferous tubules and smaller ovaries; thus, it was difficult to analyze radiation effects directly on the gametes. Therefore, we indirectly evaluated the effects of radiation on sperm and oocytes by analyzing gamete-specific genes in the surviving cells that differentiate into spermatocytes in the testes and in the oocytes that would be ovulated. We analyzed global DNA methylation patterns in the testes and gene expression levels of *Izumo1* and *Izumo1r*, which are essential for sperm and oocyte fusion in the gonads of mice, at 35 and 100 days old. These mice were irradiated with LDR or HDR before their sexual maturity at 7 days old. The global DNA methylation status in the testes was different between the control and irradiated groups. As for *Izumo1* gene expression levels in the testes, it recovered after the end of irradiation in the LDR group, while it remained low regardless of age in the HDR group. On the other hand, *Izumo1r* (*Juno*) gene expression levels in the ovary decreased with age in the control and LDR groups, and remained low in the HDR group. The study indicated that LDR and HDR in mice before sexual maturity might affect gametogenesis and fertility.

Research on possible radiation effects on human and animal gonads has shown that germ cells are highly radiosensitive, but these effects on fertility are controversial [41,42,43,44,45]. For the testes, it is generally accepted that both low- and high-dose radiation negatively affected differentiation, and eliminated spermatogonia and subsequent generations [2,3,33,46]. However, spermatogenesis was able to recover under certain circumstances after irradiation, depending on the age of when irradiation occurred and the total dose. The spermatogonial stem cell pool is hence highly likely to be affected during radiation [47,48]. In Shin et al.’s study, 7-week-old mice were irradiated to a total dose of 4 Gy [49]. Irradiation at LDR (0.7 mGy/h) did not affect spermatogonia, but HDR exposure (0.8 Gy/min) significantly increased sperm abnormalities. In another study focusing on raccoon testes, although the total amount of radiation was unknown, chronic LDR exposure associated with the Fukushima Daiichi Nuclear Power Plant (FDNPP) accident had no adverse effect on their reproductive characteristics and functions. [50]. Moreover, enhanced spermatogenesis occurred in large Japanese field mice (*Apodemus speciosus*) living in and around the FDNPP ex-evacuation zone (407–447 μGy/day) [43]. In contrast, dysregulation of methylation in mice testes after LDR and HDR was seen in our study, and it was possible that some genetic abnormality has occurred as these mice were irradiated before sexual maturity. The testes are composed of various differentiating cells, such as diploid premeiotic and meiotic cells and haploid post-meiotic cells. The germinal population is very heterogeneous, making radiation sensitivity and gene expression complex. Irradiation alters DNA methylation patterns in a tissue-specific manner, leading to a genome-wide loss of DNA methylation [32,51,52]. On the other hand, a global gain of DNA methylation is seen during cell differentiation, although there are certain loci which lose methylation in specific cell types [53]. In order to recover depleted testicular cells damaged after irradiation, we can expect an increase in cell division and differentiation, resulting in a dysregulation of DNA methylation.

In mice, spermatozoa are observed between 3 and 6 weeks old, and testicular development is completed at 11 weeks [54]. Testicular cells were likely reduced after LDR irradiation at 35 days of age as *Izumo1* expression was significantly down-regulated as compared to the control group. *Izumo1* was then subsequently up-regulated at 100 days old, possibly due to the recovery and differentiation of testicular cells. On the other hand, testicular cells including spermatogonia stem cells may have been depleted after HDR irradiation, as the subsequent recovery of *Izumo1* gene expression was not observed in our study.

In most mammals, the pool of the female germ cells in the ovary is arrested in the first meiotic prophase as germinal-vesicle-stage oocytes, and the number of follicles in the ovary gradually decreases throughout postnatal life [55,56]. Thus, as observed in the control group of our study, *Izumo1r* mRNA expression levels were downregulated with age. The ovary is also susceptible to radiation damage, but it is also difficult to determine the extent of damage. Kimler et al. showed that after whole-body gamma radiation (2.1 Gy/min) at 6 weeks old, the total number of follicles in mice decreased at 1 Gy, but there was no change at 0.1 Gy [4]. Furthermore, follicular renewal after radiation-induced depletion of oocytes has not been observed and is thought to cause premature ovarian failure, low fertility and infertility [57]. The markedly reduced *Izumo1r* gene expression in both LDR and HDR groups in our study also supports no follicular renewal after radiation-induced oocyte depletion. This study also emphasizes that if radiation exposure occurs in an early age, an increased risk of infertility can be observed.

In this study, chronic LDR exposure of young male mice showed potential gene expression recovery of genes involved in fertilization after the end of irradiation. On the other hand, LDR exposure in young female mice did not recover 75 days after exposure, suggesting that the ovaries were extremely radiosensitive. Notably, our study suggests that both LDR and HDR at a young age can cause changes in global DNA methylation patterns, which might affect gametogenesis and fertility even if gene expression is recovered. Although our results were not able to establish a relationship between radiation-induced changes in DNA methylation patterns and changes in mRNA levels of *Izumo1* and *Izumo1r*, the methylation status of their promoters will be elucidated in the future.

## 5. Conclusions

The nuclear power plant accident at Fukushima has raised the need to understand the late effects of chronic exposure to LDR. It is crucial to understand epigenetics and gene expression related to fertilization as they have important implications for reproductive health and transgenerational effects. Furthermore, these effects after long-term exposure to LDR are not as well studied in mice irradiated before sexual maturity.

From our experimental setup, we indicated that LDR can induce different DNA methylation patterns in the testes. Moreover, both HDR and LDR before sexual maturity might affect gametogenesis and fertility.

Despite our limited data, the conclusions drawn from this study can shed some light on possible effects on the gonads after radiation disasters and radiation therapy in young people. It is necessary from the viewpoint of radiation protection and the evaluation of victims’ quality of life. Further analysis will be performed in the future.

## Data Availability

All data are provided in the article.
